# Draft Genome Sequences of Clinical Isolates of Multidrug-Resistant *Acinetobacter baumannii*

**DOI:** 10.1128/genomeA.01547-16

**Published:** 2017-02-02

**Authors:** Keesha E. Erickson, Nancy E. Madinger, Anushree Chatterjee

**Affiliations:** a Chemical and Biological Engineering, University of Colorado Boulder, Boulder, Colorado, USA; b Division of Infectious Diseases, University of Colorado Anschutz Medical Campus, Aurora, Colorado, USA; c BioFrontiers Institute, University of Colorado Boulder, Boulder, Colorado, USA

## Abstract

We report here the draft genome sequences of two clinically isolated *Acinetobacter baumannii* strains. These samples were obtained from patients at the University of Colorado Hospital in 2007 and 2013 and encode an estimated 20 and 13 resistance genes, respectively.

## GENOME ANNOUNCEMENT

Pathogens in the genus* Acinetobacter* have been named as a serious threat to public health (1), with *Acinetobacter baumannii* in particular capable of increasing risk of mortality up to 70% (2). *A. baumannii* is a decidedly adaptive species, demonstrating a wealth of mechanisms for evading diverse antibiotic classes, including on horizontally transferred resistance islands in the genome (2, 3) and resistance-conferring plasmids (4). *A. baumannii* is also able to survive for extended durations on dry surfaces (5). These factors together have allowed for a high frequency of multidrug-resistant nosocomial *A. baumannii* infections worldwide, with diminishing options for treatment. Characterization of clinically isolated *A. baumannii* strains will enable development of more effective treatment strategies.

We sequenced two *A. baumannii* strains from the University of Colorado Hospital Clinical Microbiology Laboratory organism bank. Strain CU060707 was isolated from the bloodstream of a 47-year-old male in 2007, and strain CU032113 was isolated from a hardware-associated bone infection in a 44-year-old female in 2013.

Colonies were grown aerobically in cation-adjusted Mueller-Hinton broth for 16 h at 37°C. Approximately two micrograms of DNA were isolated per strain (Wizard DNA purification kit, Promega). The Nextera XT DNA library kit was used to build paired-end 250-bp libraries, which were sequenced on an Illumina MiSeq, generating 1,644,910 and 1,081,729 reads for CU060707 and CU032113, respectively. FASTQ files were filtered using the sliding window mode in Trimmomatic (6). *De novo* assembly was performed with SPAdes version 1.0.0 (7) and Rescaf version 1.0.1; then annotation was executed with Prokka version 1.0.0 (8) in BaseSpace (https://basespace.illumina.com). Antibiotic resistance genes were identified with ARG-ANNOT (9).

The draft assembly of *A. baumannii* CU060707 is 4,119,754 bp in length (94× coverage, 39% GC content) in 240 contigs with an *N*_50_ of 150,723 bp. The genome contains 56 tRNAs, seven rRNAs, one CRISPR, and 3,831 coding sequences. The 20 resistance genes detected include six aminoglycosides [*ant(3ʺ)-Ih*, *aadA1*, *aph(3ʺ)-Ia*, *armA*, *strA*, and *strB*], nine β-lactams (ADC-30, *blaA1*, *blaA2*, *mbl*, OXA-235, OXA-66, TEM-1D, a penicillin-binding protein, and a zinc-dependent hydrolase), two macrolide-lincosamide-streptogramins (*mphE*, *msrE*), one chloramphenicol (*catB8*), one sulfonamide (*sulI*), and one tetracycline (*tetB*). Most resistance genes are encoded on the genome, though OXA-235 appears to be on a plasmid, similar to GenBank CP015486.1.

The draft assembly of *A. baumannii* CU032113 is 4,182,411 bp in length (61× coverage, 39% GC content) in 322 contigs with an *N*_50_ of 59,362 bp. There are 70 tRNAs, nine rRNAs, two CRISPRs, and 3,882 coding sequences. The 13 resistance genes found include two aminoglycosides (*strA* and *strB*), eight β-lactams (ADC-76, *blaA1*, *blaA2*, *mbl*, OXA-24, OXA-65, TEM-116, and a zinc-dependent hydrolase), two chloramphenicols (*catA1* and *cmlB1*), and one sulfonamide (*sulII*). OXA-24 is the only gene not on the genome; it appears to be on a plasmid, similar to GenBank JN207494.1 (4).

We validated resistances by plating on solid media with antibiotics and found that both strains are able to grow in tetracycline, ampicillin, and chloramphenicol. CU060707 cannot grow in meropenem. CU032113 grows in meropenem, but not in gentamicin or erythromycin. Both isolates are resistant to ciprofloxacin, indicating that *gyrA* and/or *parC* are mutated (10, 11).

### Accession number(s).

This whole-genome shotgun sequencing project has been deposited in GenBank under the accession numbers LXWY00000000 (for CU060707) and LXYW00000000 (for CU032113).

## References

[1] CDC 2013 Antibiotic resistance threats in the United States, 2013. U.S. Department of Health and Human Services, CDC, Atlanta, GA.

[2] Peleg AY, Seifert H, Paterson DL 2008 *Acinetobacter baumannii*: emergence of a successful pathogen. Clin Microbiol Rev 21:538–582. doi:10.1128/CMR.00058-07. 18625687 PMC2493088

[3] Zhu L, Yan Z, Zhang Z, Zhou Q, Zhou J, Wakeland EK, Fang X, Xuan Z, Shen D, Li QZ 2013 Complete genome analysis of three *Acinetobacter baumannii* clinical isolates in China for insight into the diversification of drug resistance elements. PLoS One 8:e66584. doi:10.1371/journal.pone.0066584. 23826102 PMC3691203

[4] Azam N, Talukder T, Robinson KR, Kwon DH 2015 Dissemination and genetic Structure of carbapenemase encoding genes (bla_OXA-23_ and bla_OXA-24_) in *Acinetobacter baumannii* from Southern Texas. Adv Microbiol 4:457–468. doi:10.4236/aim.2015.56047.

[5] Viehman JA, Nguyen MH, Doi Y 2014 Treatment options for carbapenem-resistant and extensively drug-resistant *Acinetobacter baumannii* infections. Drugs 74:1315–1333. doi:10.1007/s40265-014-0267-8. 25091170 PMC4258832

[6] Bolger AM, Lohse M, Usadel B 2014 Trimmomatic: a flexible trimmer for Illumina sequence data. Bioinformatics 30:2114–2120. doi:10.1093/bioinformatics/btu170. 24695404 PMC4103590

[7] Bankevich A, Nurk S, Antipov D, Gurevich AA, Dvorkin M, Kulikov AS, Lesin VM, Nikolenko SI, Pham S, Prjibelski AD, Pyshkin AV, Sirotkin AV, Vyahhi N, Tesler G, Alekseyev MA, Pevzner PA 2012 SPAdes: a new genome assembly algorithm and its applications to single-cell sequencing. J Comput Biol 19:455–477. doi:10.1089/cmb.2012.0021. 22506599 PMC3342519

[8] Seemann T 2014 Prokka: rapid prokaryotic genome annotation. Bioinformatics 30:2068–2069. doi:10.1093/bioinformatics/btu153. 24642063

[9] Gupta SK, Padmanabhan BR, Diene SM, Lopez-Rojas R, Kempf M, Landraud L, Rolain JM 2014 ARG-ANNOT, a new bioinformatic tool to discover antibiotic resistance genes in bacterial genomes. Antimicrob Agents Chemother 58:212–220. doi:10.1128/AAC.01310-13. 24145532 PMC3910750

[10] Vila J, Ruiz J, Goñi P, Jimenez de Anta TJ 1997 Quinolone-resistance mutations in the topoisomerase IV parC gene of *Acinetobacter baumannii*. J Antimicrob Chemother 39:757–762. doi:10.1093/jac/39.6.757. 9222045

[11] Vila J, Ruiz J, Goñi P, Marcos A, Jimenez de Anta TJ 1995 Mutation in the gyrA gene of quinolone-resistant clinical isolates of *Acinetobacter baumannii*. Antimicrob Agents Chemother 39:1201–1203. doi:10.1128/AAC.39.5.1201. 7625818 PMC162713

